# Clinical and Cardiovascular Magnetic Resonance Findings in Endurance Athletes With Symptomatic Stroke

**DOI:** 10.1016/j.jaccas.2026.107352

**Published:** 2026-03-25

**Authors:** Bradley S. Chambers, Wasim Javed, Saad Fyyaz, Ben Mercer, Chin Y. Soo, Michael Papadakis, Sanjay Sharma, Peter P. Swoboda

**Affiliations:** a Leeds Institute for Cardiovascular and Metabolic Medicine, University of Leeds, Leeds, United Kingdom; b Cardiovascular and Genomics Research Institute, City St George's, University of London, London, United Kingdom; c Leeds Teaching Hospitals Trust, Leeds, United Kingdom

**Keywords:** atrial fibrillation, cardiovascular magnetic resonance, exercise, hypertension, stroke

## Abstract

**Background:**

Stroke in athletes is believed to be rare given fewer traditional stroke risk factors in this population, although there is a distinct lack of data. We present a case series of 6 veteran endurance athletes who experienced symptomatic strokes and underwent cardiovascular magnetic resonance imaging.

**Case Summary:**

Underlying causes of the stroke were established in 5 athletes: atrial fibrillation (n = 2), likely apical hypertrophic cardiomyopathy with thrombus, patent foramen ovale, and carotid artery dissection. Nonischemic myocardial fibrosis was highly prevalent, while exercise-induced hypertension was present in approximately half of the cases.

**Discussion:**

Our findings illustrate a range of etiologies causing stroke in athletes. They also highlight weaknesses with stroke risk-stratification methods in athletes with atrial fibrillation, along with possible modifiable risk factors including masked hypertension and exercise-induced hypertension. Our cases highlight the role of cardiovascular magnetic resonance in detecting the etiology of stroke and propose myocardial fibrosis as a potential novel marker of stroke risk in veteran endurance athletes.

## Visual Summary

**Figure:**
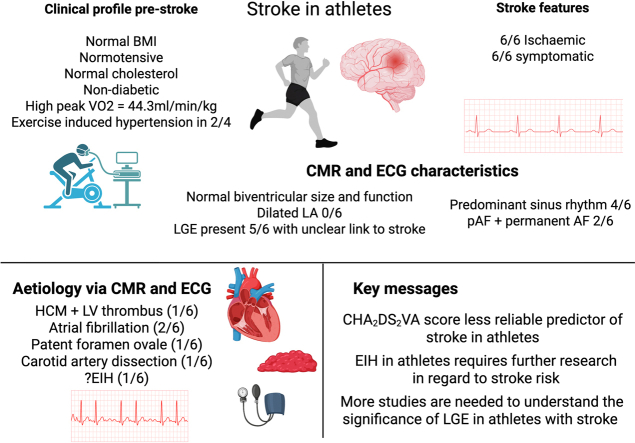

Regular aerobic exercise reduces the risk of cardiovascular disease. However, certain older athletes paradoxically exhibit an increased prevalence of specific cardiovascular disorders including hypertension, atrial fibrillation (AF), and coronary atherosclerosis. Among the general population, these conditions are strongly associated with stroke. However, there is a paucity of data related to the incidence of stroke in athletes, particularly endurance athletes, as well as the clinical characteristics of those athletes who experience stroke. Here, we present a case series of 6 previously otherwise healthy veteran endurance athletes who experienced symptomatic ischemic stroke. The demographic and athletic history of the participants is shown in Table 1, and a summary of their imaging characteristics is shown in Table 2.

**Take-Home Messages**

• The CHA_2_DS_2_-VA score in athletes with atrial fibrillation may not correlate with stroke risk.
• Exercise-induced hypertension in athletes needs to be better understood, particularly regarding potential risk in stroke.
• Further research is needed to understand the significance of late gadolinium enhancement in athletes with respect to stroke.

**Table 1:** Demographics and Athletic History of the Study Participants

Case	Age at Stroke (y)	CHA_2_DS_2_-VA Before Stroke	Sex	BMI (kg/m^2^)	Ethnicity	Sport	EIH (mm Hg)	Peak VO_2_ (mL/min/kg)	Weekly Exercise (h)	Exercise History (y)	Suspected Stroke Etiology
1	82	2	M	22	Caucasian	Runner	a	36.3	3	58	Permanent AF
2	66	1	M	25.2	Caucasian	Cyclist	206	46.2	15	44	pAF
3	52	0	M	22.3	Caucasian	Runner	299	51.3	9	40	Cryptogenic, HTN-related?
4	57	0	M	22.5	Caucasian	Runner, triathlon	a	73^b^	13	34	Apical HCM
5	63	0	M	23.5	Caucasian	Runner	181	41.3	6	25	Carotid artery dissection
6	59	0	F	20.6	Caucasian	Runner	200	46.4	6.5	13	PFO

AF = atrial fibrillation; BMI = body mass index; EIH = exercise-induced hypertension; HCM = hypertrophic cardiomyopathy; HTN = hypertension; pAF = paroxysmal atrial fibrillation; PFO = patent foramen ovale; VO_2_ = inspired oxygen concentration.

a Not tested.

b Attained at younger age.

**Table 2:** CMR Characteristics of the Study Participants

Case	LVEDVi (mL/m^2^)	LVESVi (mL/m^2^)	LVEF (%)	LV Mass (g)	RVEDVi (mL/m^2^)	RVESVi (mL/m^2^)	RVEF (%)	LA Area (cm^2^)	LGE	LGE Pattern
1	74	22	71	141	67	18	73	29	1	RVIP
2	99	43	56	126	93	35	62	28	1	NI LGE, basal to mid lateral + RVIP
3	82	34	58	197	76	29	61	28	1	RVIP
4	109	34	69	140	90	40	55	20	1	Transmural apical cap and RVIP
5	79	22	72	178	68	13	81	24	1	NI LGE, mid inferolateral
6	104	35	67	90	109	48	56	20	0	—

LA = left atrium; LGE = late gadolinium enhancement; LV = left ventricle; LVEDVi = left ventricular end-diastolic volume index; LVESVi = left ventricular end-systolic volume index; LVEF = left ventricular ejection fraction; NI = nonischemic; RVEDVi = right ventricular end-diastolic volume index; RVESVi = right ventricular end-systolic volume index; RVEF = right ventricular ejection fraction; RVIP = right ventricular insertion point.

## Case 1

**Figure 1:**
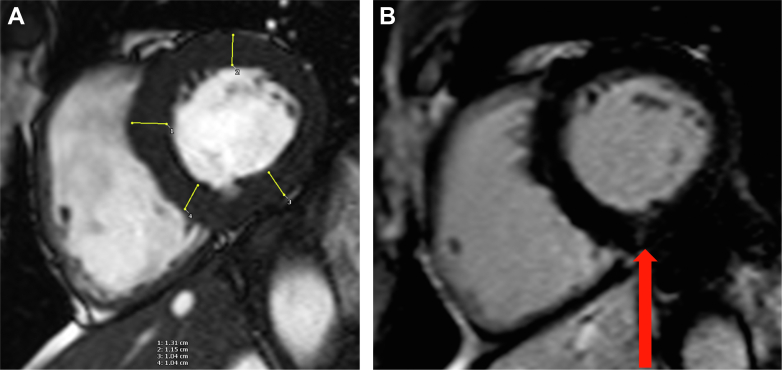
CMR Images for the Patient in Case 1 (A) Eccentric LVH with maximum wall thickness of 13 mm at the basal slice level, measured using yellow lines in CVI42 (Circle Cardiovascular Imaging Inc). (B) RV insertion point fibrosis (arrow). CMR = cardiovascular magnetic resonance; LVH = left ventricular hypertrophy; RV = right ventricular.

An 82-year-old previous national level male runner had exercised for 3 hours per week for 58 years and demonstrated 133% of predicted peak inspired oxygen concentration (VO_2_) on exercise testing. A 12-lead electrocardiogram (ECG) showed normal sinus rhythm. He was found to have permanent AF on Holter monitoring 10 months prior to presentation. His CHA_2_DS_2_-VA score was 2 (scored for age only), for which he was taking anticoagulants. Despite anticoagulation, he experienced a left-sided partial anterior circulation stroke, with AF attributed as the underlying cause. After the stroke, he was initiated on treatment for hypertension. As shown in Figure 1, cardiovascular magnetic resonance (CMR) imaging showed normal biventricular size and function (left ventricular ejection fraction [LVEF]: 71%), left atrial (LA) size at the upper limit of normal (29 cm^2^), and left ventricular (LV) maximum wall thickness of 13 mm at the basal septum, demonstrating eccentric hypertrophy with LV mass of 141 g.

This case demonstrates a case of stroke due to AF despite anticoagulation. The patient likely had long-standing occult hypertension, which was only detected poststroke. Hypertension is one of the most commonly diagnosed conditions on preparticipation screening, with a predilection in older athletes. Furthermore, many athletes may have masked hypertension or nocturnal hypertension. Hypertension is also a well-established risk factor for stroke in the general population, where it is believed to be the most important modifiable risk factor. While moderate physical activity reduces the risk of incident stroke, the REGARDS study showed this was likely mediated via reduction of traditional risk factors including hypertension.^1^ Therefore, athletes with hypertension may not reap the stroke-reducing benefits associated with regular exercise. This case therefore highlights the need to actively screen for traditional modifiable stroke risk factors, in particular masked hypertension, in veteran athletes with AF.

## Case 2

**Figure 2:**
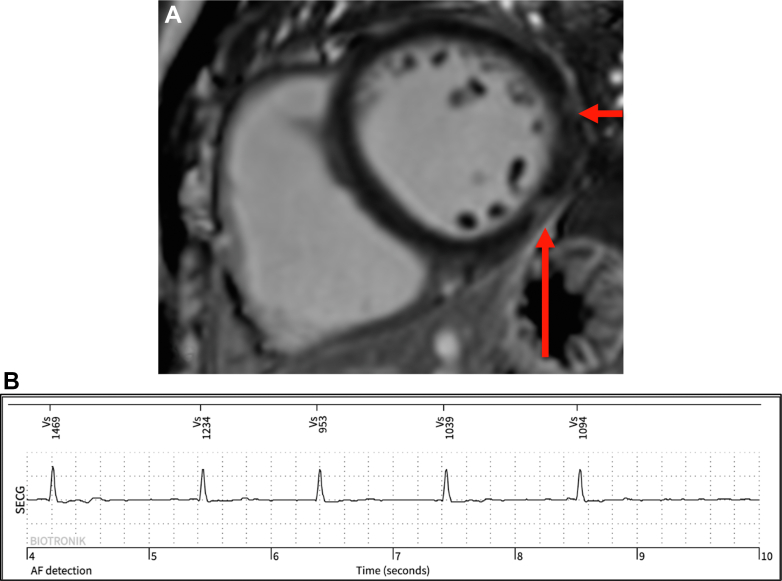
CMR Images and Implantable Loop Recorder Evidence of AF for the Patient in Case 2 (A) Basal to mid–lateral wall myocardial fibrosis (arrows) and RV insertion point fibrosis. (B) AF episode on Biotronik implantable loop recorder. AF = atrial fibrillation; CMR = cardiovascular magnetic resonance; RV = right ventricular.

A 66-year-old lifelong competitive male cyclist presented with transient loss of vision followed by left-sided homonymous hemianopia. MRI subsequently showed partial occipital circulation stroke. The participant already had an implantable loop recorder in situ as part of a research study, where initial 12-lead ECG showed sinus rhythm with first-degree atrioventricular block (PR interval: 262 ms). The implantable loop recorder was interrogated to identify new-onset paroxysmal AF corresponding to his symptoms. Furthermore, there were episodes of asymptomatic profound bradyarrhythmia, with sinus bradycardia <30 beats/min. AF was attributed as the underlying etiology, and anticoagulation was commenced. As shown in Figure 2, CMR demonstrated normal biventricular size and function (LVEF: 56%) and LA area at the upper limit of normal (28 cm^2^). On CMR with late gadolinium enhancement (LGE), nonischemic fibrosis of the basal to mid inferolateral segments was present. On exercise testing, exercise blood pressure was 206/97 mm Hg, which is considered to be at the upper limit of normal.

This case represents a first presentation of AF resulting in stroke. This was despite a CHA_2_DS_2_-VASc of only 1 based solely on age. Data regarding the risk of stroke in athletes with AF are limited. The Birkebeiner aging study (BiAS) demonstrated that veteran athletes with AF (mean age 67 years, 91% male) were almost twice as likely to experience a stroke than athletes without AF.^2^ However, this risk was lower when compared with nonathletes with AF.^3^ The AFLETES study surveyed 942 athletes (84% male) and found that athletes who reported AF were 4 times more likely to have a stroke despite a low CHA_2_DS_2_-VA score.^4^ Therefore, the risk of stroke may be underestimated by traditional scoring systems. This case highlights the limitations of traditional stroke risk-stratification methods in athletes and the importance of early detection of AF due to its potential to cause stroke at first onset.

## Case 3

**Figure 3:**
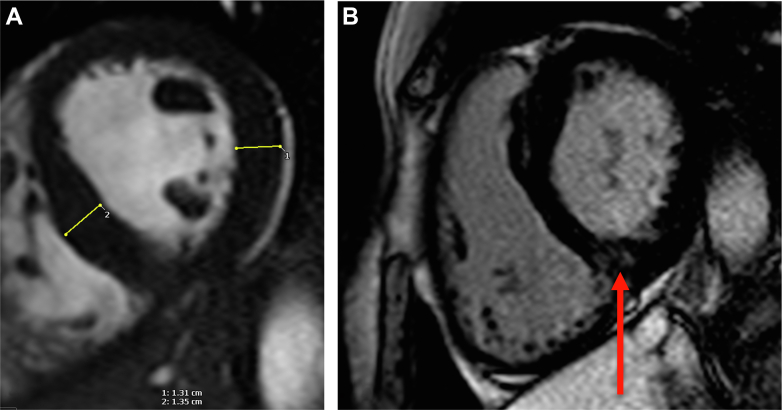
CMR Images for the Patient in Case 3 (A) LV wall thickness at mid-LV level shows concentric hypertrophy of 13 and 14 mm in the septal and lateral walls. (B) RV insertion point fibrosis (arrow). CMR = cardiovascular magnetic resonance; LV = left ventricular; RV = right ventricular.

A 52-year-old national level male runner who exercised for 9 hours per week for the prior 40 years demonstrated 137% of predicted peak VO_2_. Despite normal resting blood pressure, he exhibited significant exercise-induced hypertension (EIH) (299/154 mm Hg) on exercise testing. This athlete presented with signs and symptoms consistent with a clinical stroke. A 12-lead ECG showed normal sinus rhythm and left-axis deviation only, and Holter monitoring did not detect AF. Hypertension was diagnosed on admission for his stroke, for which he received appropriate treatment. As shown in Figure 3, CMR demonstrated normal biventricular size and function (LVEF: 58%) with concentric LV hypertrophy (13 mm in septal and 14 mm in lateral walls) with elevated LV mass (197 g) and LA area at the upper limit of normal (28 cm^2^). Inferior right ventricular insertion point fibrosis was present.

A clear etiology was not identified, and therefore the cause was deemed cryptogenic. However, hypertension was suspected to have played a role given the elevated blood pressure readings on admission alongside a strong family history. This athlete also demonstrated marked EIH of 299/154 mm Hg. EIH increases the risk of developing future overt hypertension in athletes, but its impact on stroke in athletes has not been studied. In sedentary individuals, EIH is not associated with stroke.^5^ However unlike athletes, these participants do not undertake several hours of exercise per week. Furthermore, the majority of the participants in this study had resting hypertension, and thus athletes with EIH may experience far greater sudden blood pressure fluctuations during exercise. While the relationship between blood pressure and cerebral blood flow is complex, middle cerebral artery velocity increases in line with blood pressure during high-resistance, high-intensity exercise.^6^ This suggests that the exaggerated blood pressure response to exercise may directly transmit to the cerebral vasculature. Given this athlete had an extensive training history, it is plausible that repeated bouts of EIH may have contributed to his stroke.

## Case 4

**Figure 4:**
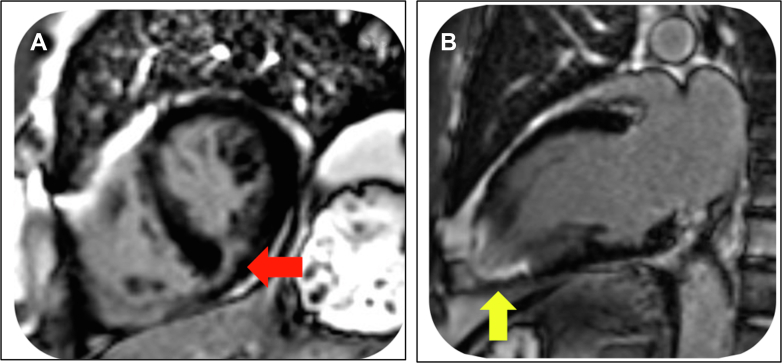
CMR Images for the Patient in Case 4 (A and B) HCM with transmural LGE in inferior apical wall with adjacent thrombus visible. LGE (red and yellow arrows). CMR = cardiovascular magnetic resonance; HCM = hypertrophic cardiomyopathy; LGE = late gadolinium enhancement.

A 57-year-old elite level male runner/triathlete presented with symptomatic stroke and was found to have right-sided occipital infarct. Holter monitoring was performed, which did not reveal AF. A 12-lead ECG showed anterolateral T-wave inversion; therefore he underwent CMR (Figure 4). This demonstrated apical hypertrophic cardiomyopathy (HCM) with apical LV hypertrophy (13 mm) and systolic cavity obliteration. There was also apical transmural fibrosis with adjacent thrombus. LV thrombus was considered as the most likely primary mechanism for stroke, and he subsequently underwent anticoagulation treatment.

HCM is an important cause of sudden cardiac death in younger athletes. Those with an apical variant are less likely to report a positive family history than those with classic HCM. Furthermore, apical LV hypertrophy may not be detected by echocardiography. Therefore, conventional screening strategies may not identify apical HCM. CMR has increased sensitivity for detecting apical HCM, and this case highlights the advantage of performing CMR in athletes who experience stroke, especially in the presence of ECG abnormalities. Here, CMR not only detected the presence of apical HCM but also LV thrombus and extensive scar, which helped diagnose and further risk-stratify the athlete.

## Case 5

**Figure 5:**
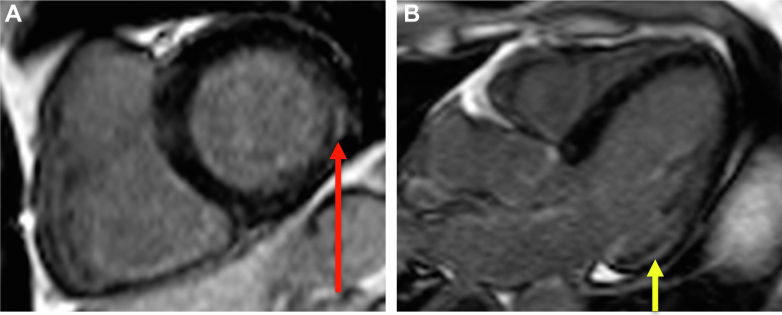
CMR Images for the Patient in Case 5 (A and B) Basal inferolateral LGE (red and yellow arrows) seen on short-axis and apical 3-chamber views. CMR = cardiovascular magnetic resonance; LGE = late gadolinium enhancement.

A 63-year-old male runner who trained for 6 hours per week for the previous 25 years attained 134% of predicted peak VO_2_ on exercise testing and had nonelevated blood pressure response to exercise. A 12-lead ECG showed normal sinus rhythm and left-axis deviation, however paroxysmal supraventricular tachycardia episodes were noted on Holter monitoring, with the absence of AF. He presented with persistent headache radiating from the neck, and a carotid artery dissection was identified. Figure 5 shows CMR which demonstrated normal biventricular size and function (LVEF: 72%) and normal LA size. Nonischemic fibrosis was noted in the basal inferolateral wall.

Extracardiac causes are an important cause of strokes in athletes. High-impact sports involving head and/or neck trauma along with weightlifting are believed to increase the risk of hemorrhagic stroke and vascular dissection, particularly in those with a predisposition such as an intracerebral or cervical vascular defects.^7^ This case is unusual, as the runner had not sustained neck trauma. However, there are multiple case reports of similar presentations involving noncontact sport in endurance athletes.^8^ Therefore, cervical vascular defects should be considered as a cause for stroke in endurance athletes when there is clinical suspicion and especially in the absence of cardiac pathology.

## Case 6

A 59-year-old female runner who trained for 6.5 hours per week for 30 years and attained 153% of predicted peak VO_2_ and an exaggerated blood pressure response during exercise testing (200/90 mm Hg) experienced a stroke. This was after 13 years of training and was immediately preceded by a period of altitude training. A patent foramen ovale (PFO) was discovered on echocardiogram and was considered the underlying etiology. CMR demonstrated mildly dilated biventricular size with normal function. A 12-lead ECG demonstrated sinus rhythm, left-axis deviation, and T-wave inversion in leads V_1_ and V_2_. Her LA size was normal, and no LGE was present.

PFOs are believed to be present in up to 25% of the general population. The risk of stroke posed from a PFO is controversial. However, vigorous exercise has been found to increase the likelihood of developing a PFO-related stroke. This may be due to raised intracardiac pressure leading to greater right-to-left shunting or Valsalva-type exercises, which are both believed to increase the risk of PFO-related stroke.^9^ This athlete's EIH may have increased the risk of PFO-related stroke. Given the global emerging trends in percutaneous PFO closures and the potential benefit in certain patient cohorts, PFO-related stroke should be considered in athletes who experience stroke of an unknown etiology after thorough diagnostic work-up.

## Nonischemic Fibrosis: A Marker of Risk?

An increased prevalence of nonischemic myocardial fibrosis has been demonstrated among seemingly healthy athletes, particularly in older, male athletes who chronically perform high-intensity exercise.^10^ While this has been shown to be associated with ventricular arrhythmia in veteran male athletes, there are no data investigating whether nonischemic fibrosis is implicated in stroke in athletes.

In this case series, 5 of the 6 athletes who experienced stroke were found to have nonischemic fibrosis. Although the total number of athletes included in this series is too few to draw any substantial conclusions, it is possible that nonischemic fibrosis may be an indicator of increased risk of ischemic stroke. This may simply represent coexisting pathology such as masked hypertension, EIH, or concealed cardiomyopathy, however it may be possible to identify myocardial fibrosis before these cardiac disorders are clinically, overtly apparent and therefore could be a useful tool in the risk stratification of stroke in endurance athletes.

## Conclusions

Despite lower rates of traditional risk factors, lifelong endurance athletes still have residual risk of stroke. The etiology is heterogeneous and may specifically include AF, hypertension, and EIH. When AF is present, traditional stroke risk stratification may underestimate the true risk of stroke. Nonischemic myocardial fibrosis may be a novel marker of stroke risk in veteran athletes, and therefore CMR may play a role in both detecting stroke etiology and risk stratification. However, more prospective research is needed to further investigate this.

**Visual Summary:**
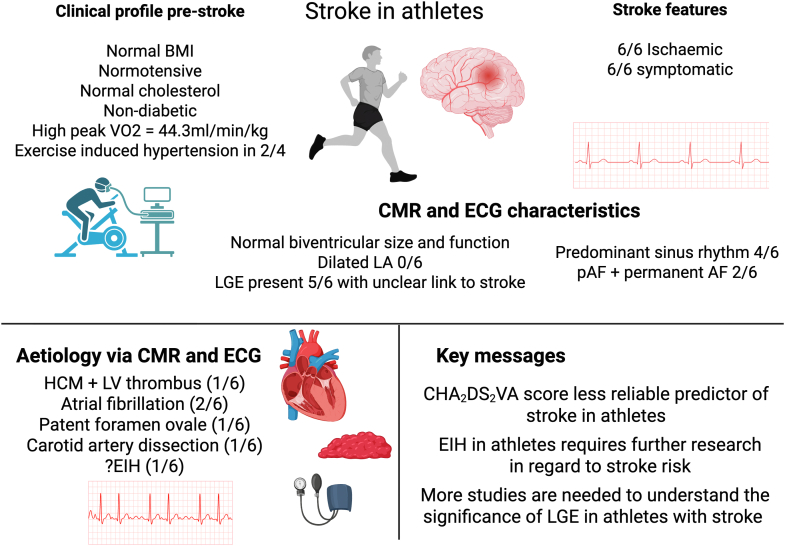
Stroke in Athletes Case series of stroke in athletes: clinical and CMR characteristics, etiologies, and key messages. Created in BioRender. Soo CY (2026), https://BioRender.com/0zmqhwn. AF = atrial fibrillation; BMI = body mass index; CMR = cardiovascular magnetic resonance; ECG = electrocardiogram; EIH = exercise-induced hypertension; HCM = hypertrophic cardiomyopathy; LA = left atrium; LGE = late gadolinium enhancement; LV = left ventricle; pAF = paroxysmal atrial fibrillation; VO_2_ = inspired oxygen concentration.

## Funding Support and Author Disclosures

This research is supported by the National Institute for Health Research Leeds Biomedical Research Centre (NIHR203331). Dr Swoboda was funded by BHF award FS/CRA22/23034. Mr Chambers has received travel expenses from Novo Nordisk. Dr Papadakis has received grant support from Cardiac Risk in the Young charity and honoraria from Bristol Myers Squibb. Dr Sharma has received consulting fees from football clubs in the professional football leagues. All other authors have reported that they have no relationships relevant to the contents of this paper to disclose.
